# Natural killer cells in preeclampsia: an overview of current research

**DOI:** 10.3389/fimmu.2026.1734660

**Published:** 2026-08-18

**Authors:** Yuchao Zhang, Hang Su, Yipeng Zhang, Qichun Chen, Yuhan Meng, Cuijuan Zhang, Xuehui Bi, Qinghua Li, Weiwei Yang

**Affiliations:** 1 Department of Obstetrics, Affiliated Hospital of Shandong Second Medical University, Weifang, China; 2 School of Life Science and Technology, Shandong Second Medical University, Weifang, China; 3 School of Public Health, Shandong Second Medical University, Weifang, China; 4 Center of Reproductive Medicine, Affiliated Hospital of Shandong Second Medical University, Weifang, China

**Keywords:** preeclampsia, natural killer cells, placenta, maternal‑fetal interface, trophoblast, bibliometrics

## Abstract

**Background:**

Preeclampsia is a pregnancy disorder endangering maternal and fetal health, often accompanied by complications such as hypertension and proteinuria. Natural killer cells are indispensable in the occurrence and development of preeclampsia. Up to now, there has been no bibliometric study on NK cells and preeclampsia. Thus, we conducted a bibliometric analysis based on a systematic literature retrieval strategy.

**Methods:**

Publications related to NK cells and preeclampsia were retrieved from the Web of Science Core Collection and PubMed databases. CiteSpace and VOSviewer were applied to analyze publication trends, collaboration networks, co-citation patterns, and research hotspots.

**Results:**

The findings indicated that the prevailing research focal points in this domain predominantly encompass the maternal-fetal interface in preeclampsia, inflammatory mechanisms, immune mechanisms, and other related areas. Implantation, extracellular vesicles, and oxidative stress represent the emerging hotspots in this field in recent years.

**Conclusion:**

This study has systematically summarized the future trends in preeclampsia and natural killer cells research through bibliometrics, highlighted the current research frontiers and development trends, and provided valuable references for researchers in this field.

## 1 Introduction

Preeclampsia (PE), a pregnancy-specific syndrome threatening maternal and fetal health, contributes to the deaths of approximately 76,000 pregnant women and over 500,000 fetuses and infants annually worldwide (1). Patients with PE not only experience physical burdens such as hypertension and maternal organ damage (proteinuria, edema, dizziness) occurring after 20 weeks of gestation (2), but they and their offspring also face increased long-term risks of cardiovascular, renal, metabolic, and endocrine diseases (3–5).

Emerging studies indicate a close association between the occurrence of PE and natural killer (NK) cells. NK cells can be broadly classified based on their function and location into decidual natural killer (dNK cells, also known as uterine natural killer [uNK cells]) and peripheral blood natural killer (pNK) cells, with each subtype playing distinct roles. In patients with PE, the number of pNK cells is increased, accompanied by enhanced cytotoxicity and activity, while their ability to synthesize and secrete vascular endothelial growth factor (VEGF) is declined. In contrast, dNK cells are the most abundant immune cells at the maternal-fetal interface during early pregnancy and placenta formation. These cells are critical for secreting cytokines that promote trophoblast growth, differentiation, and invasion, facilitate the remodeling of spiral arteries, and maintain the delicate balance of angiogenesis and maternal-fetal immune tolerance (6, 7).

Bibliometrics is a systematic approach to analyze scientific publications, employing quantitative and qualitative methods to identify information from publications on specific topics. The methodology allows the evaluation of various information articles characteristics while simultaneously summarizing the current research landscape, identifying hotspots, and delineating frontiers within a specific field (8–10). When integrated with visual analysis, bibliometrics transforms into a powerful tool, empowering researchers to efficiently acquire and synthesize information on research advances (11). Therefore, we performed a bibliometric analysis based on a systematic literature retrieval strategy to characterize the development trends, knowledge structure, and emerging hotspots of NK cell-related research in PE.

## 2 Materials and methods

### 2.1 Data collection

This study was designed as a bibliometric analysis based on a systematic literature retrieval strategy. Unlike conventional systematic reviews that synthesize clinical evidence or perform meta-analysis, this study aimed to quantitatively evaluate the development trends, knowledge structure, and research hotspots of NK cell-related studies in PE.

**Figure 1:**
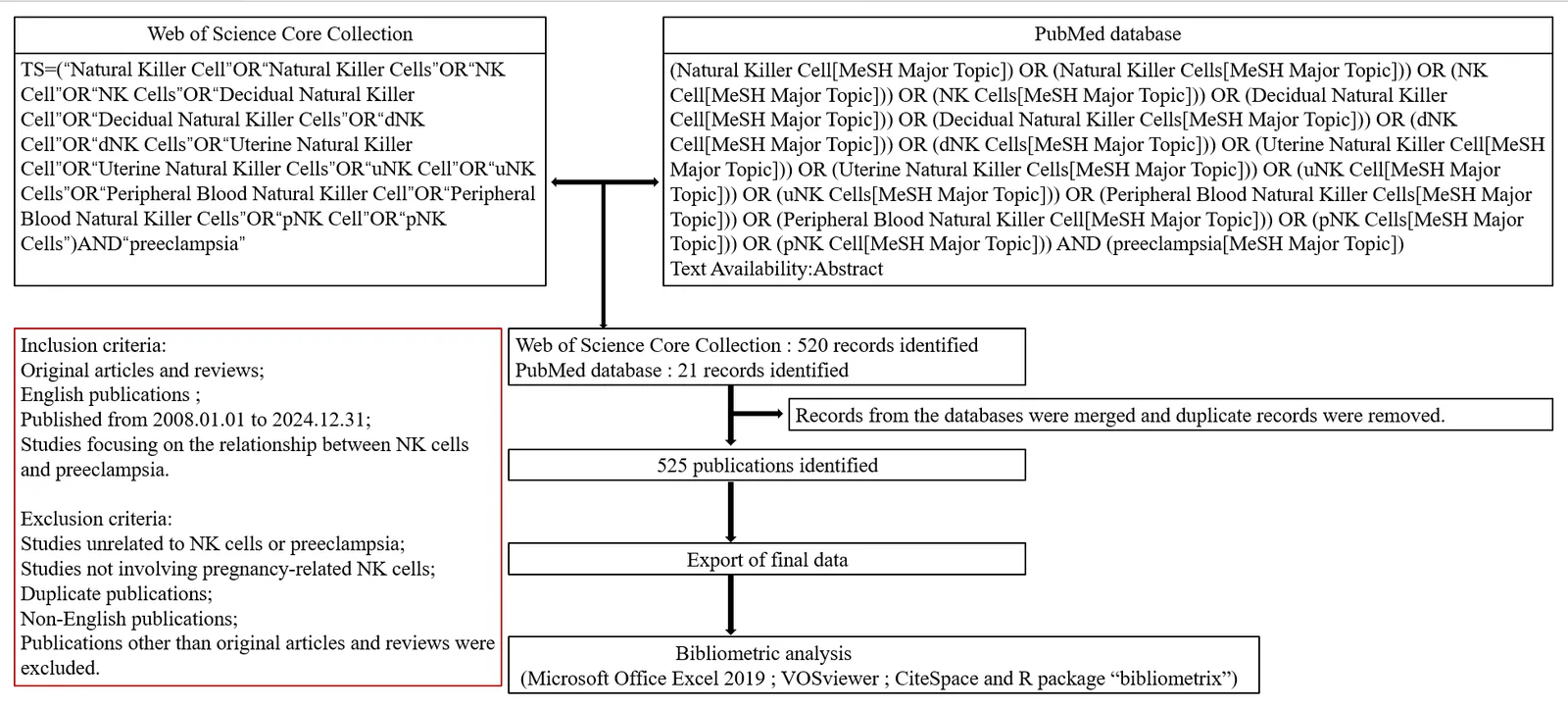
This is a flow chart of data acquisition in the study of NK cells and PE.

A comprehensive literature search was conducted on October 19, 2025, in the Web of Science Core Collection (WoSCC) and PubMed databases. The search strategy is shown in **Figure 1**, with the document types restricted to original articles and reviews. Initially, a total of 541 records were retrieved.

Two researchers (Yuchao Zhang and Hang Su) independently screened each publication for relevance based on predefined eligibility criteria. Studies were considered eligible if they investigated the relationship between NK cells and PE. Publications involving pregnancy-associated NK cell populations, including pNK cells and dNK cells, were included. In addition, studies focusing on NK cell-related characteristics, such as NK cell functional activity, cytokine production, immune regulatory functions, and molecular mechanisms underlying NK cell involvement in PE, were considered eligible for inclusion.

Publications were excluded if they were unrelated to NK cells or PE, did not involve pregnancy-associated NK cells, represented duplicate publications, were published in languages other than English, or belonged to publication types other than original articles and reviews, including conference abstracts, editorials, letters, and commentaries.

During the screening process, any disagreements between the two researchers were resolved through discussion. If consensus could not be reached, a third researcher (Yipeng Zhang) reviewed the article and made the final decision. After applying the predefined eligibility criteria and removing ineligible publications, a total of 525 articles were ultimately included in the bibliometric analysis.

The retrieved publications were not analyzed for clinical outcome synthesis but were subjected to bibliometric analysis.

### 2.2 Data analysis

The collected publications were analyzed and visualized using a combination of tools, including VOSviewer (v1.6.20.0), CiteSpace (v6.4.R1), Excel (v2019), and the R-base package “bibliometrix” (v4.5.0).

VOSviewer is able to process massive data and conduct visual analysis, displaying knowledge architectures and research directions (12). It is often used for visualizing collaboration networks, co-citation networks, and co-occurrence networks (13). This advantage enables researchers to intuitively and comprehensively understand the complex relationships among publications, significantly improving research efficiency. We used its advantages to explore the target publications.

CiteSpace is a publication visualization and analysis software. Compared with other tools, it is able to deeply mine various types of information from relevant publications. This advantage helps to understand the collaborative relationships, research hotspots, and potential trends within a specific field (14). In this study, CiteSpace visually presents the basic research context and hot frontiers related to NK cells and PE.

Based on the R package “bibliometrix”, we analyzed the trends of hot topics and keywords. Compared with other tools, it can analyze complex data and accurately predict changes in research hotspots. **Figure 1** shows the flow chart of this study. In addition, we used Microsoft Office Excel for quantitative analysis of some data.

## 3 Results

### 3.1 Number of publications per year from 2008 to 2024

**Figure 2:**
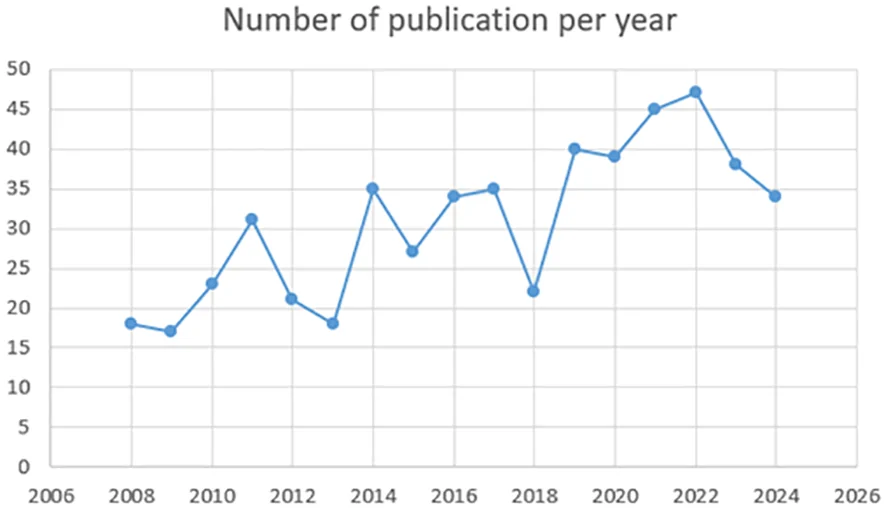
Number of published articles per year from 2008 to 2024.

The research directions and progress in a field can be judged by the number of publications over time (15). **Figure 2** demonstrates the annual publications count has remained consistently above 15 since 2008, indicating a growing interest in this field. In 2020 and 2022, 46 papers were published. Over the past decade, the number of annual publications has remained around 25, reflecting a relative stability in research activity. Meanwhile, citations have shown a steady annual growth trend.

### 3.2 Country/region analysis

**Figure 3:**
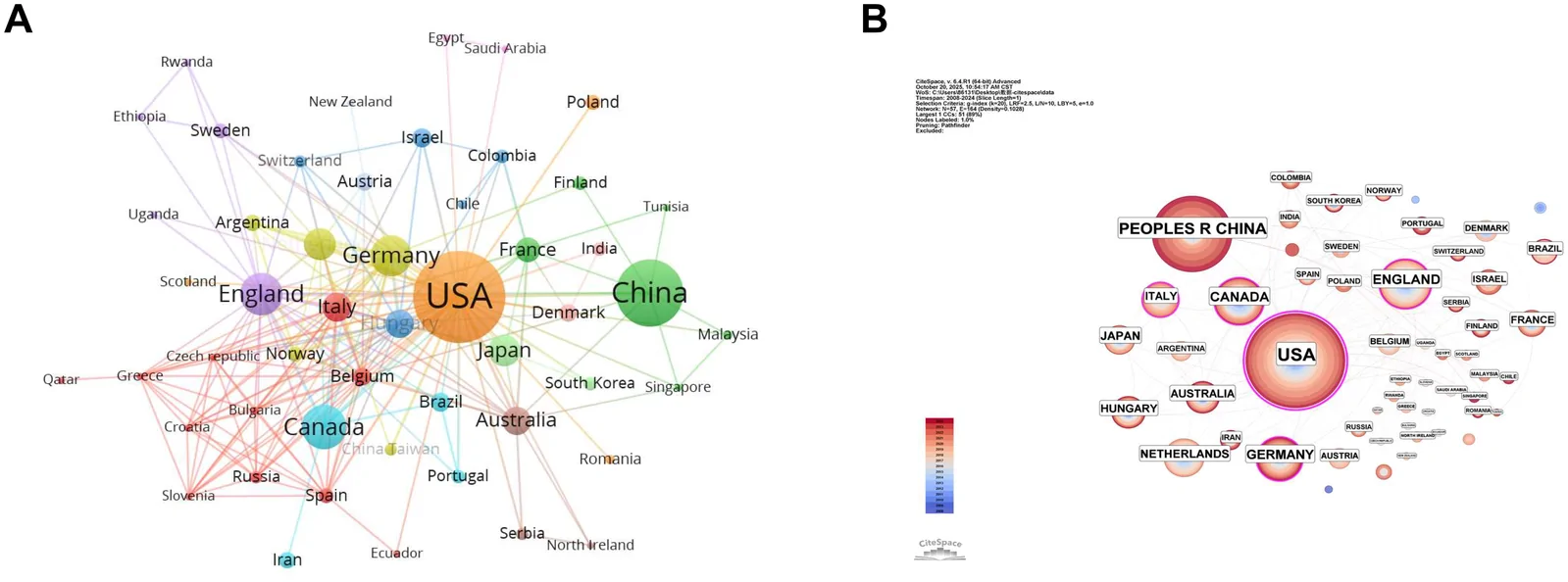
Cooperation map of countries regions in NK cells and PE. **(A)** A visual map for VOSviewer network. **(B)** A visual map for CiteSpace network.

**Table 1** shows that the USA, China, and Canada rank top three in the number of publications, accounting for 32.57%, 18.85%, and 8.95% of the total publications, respectively. In addition, five countries have a centrality ≥0.1: USA (0.84), Canada (0.16), England (0.19), Germany (0.19), and Italy (0.16). This highlights their significant positions in this field. Based on VOSviewer, we analyzed the country/region distribution network of publications, and standardized by the association strength method. The results showed that international collaboration has made significant contributions to the research on NK cells and PE. **Figure 3A** reveals that transnational research cooperation exhibits remarkable geographical characteristics: USA has formed a stable core collaboration circle with China, Canada, England, and Germany. USA closely collaborates with countries such as India, Japan, the Netherlands, Korea, Australia, France, Italy, Brazil, and Poland; China maintains close cooperation with the USA, Australia, France, Finland, Malaysia, and Tunisia; Canada frequently collaborates with the Netherlands, Austria, Belgium, Italy, Brazil, and Iran; and England collaborates with Belgium, Italy, Spain, Greece, the Netherlands, Austria, Turkiye, Sweden, Norway, and other countries. Germany cooperates with Japan, Australia, Finland, Belgium, Sweden, Korea, Serbia, and other nations. Italy cooperates with countries such as Japan, Spain, Canada, France, Israel, Switzerland, Hungary, and Belgium.

**Table 1:** Top 10 productive countries/regions in NK cells and PE.

Rank	Country	Documents	Citations	Total link strength	% of 525
1	USA	171	10590	101	32.57%
2	China	99	2609	15	18.85%
3	Canada	47	2866	26	8.95%
4	England	44	4198	42	8.30%
5	Germany	41	1394	43	7.80%
6	Netherlands	26	1960	22	4.95%
7	Japan	26	2233	10	4.95%
8	Italy	22	1154	28	4.10%
9	Hungary	21	920	29	4%
10	Australia	21	1187	22	4%

CiteSpace uses centrality to demonstrate the importance of a country in this field. According to the analysis results of CiteSpace, the nodes with purple circles in **Figure 3B** represent countries with high centrality.

### 3.3 Authors and research institutions analysis

**Figure 4:**
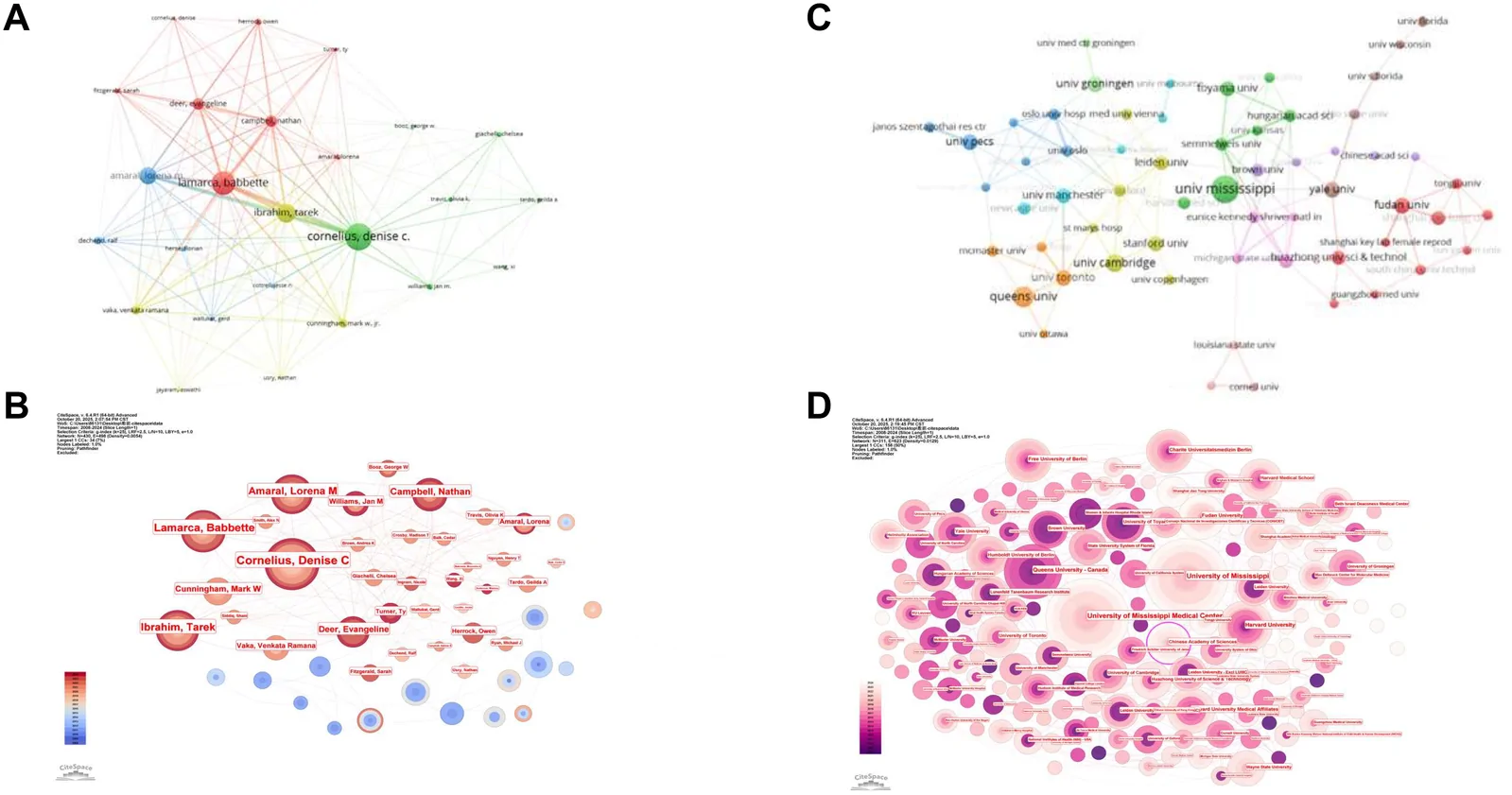
Collaboration networks among authors and among institutions in NK cells and PE. **(A)** A visual map for VOSviewer networks among authors. **(B)** A visual map for CiteSpace network among authors. Collaboration networks among authors and among institutions in NK cells and PE. **(C)** A visual map for VOSviewer network among institutions. **(D)** A visual map for CiteSpace network among institutions.

Cornelius DC from the University of Mississippi ranks first in the number of publications. The second and third places are Lamarca B and Ibrahim T (**Table 2**). The co-authorship analysis based on VOSviewer (**Figure 4A**) shows that different clusters reflect the collaborative connections among author groups. Cornelius DC closely collaborated with Baik, Cedar; Booz, George W; Giachelli, Chelsea; Williams, Jon Michael; Travis, Olivia K; Tardo, Gelida A; Williams, Jan M; Shields, Corbin A; Mccalmont, Maggie; and Wang, Xi. Lamarca B worked closely with Booz, George W; Shields, Corbin A; Williams, Jan M; Amaral, Lorena; Campbell, Nathan; Deer, Evangeline; Elfarra, Amil T; Fitzgerald, Sarah; Herrod, Owen; Dechend, Raif; and Herse, Florian. Ibrahim T frequently collaborated with Booz, George W; Shields, Corbin A; Williams, Jan M; Campbell, Nathan; Elfarra, Amil T; Fitzgerald, Sarah; Ussy, Nathan; Turner, Ty; Dechend, Raif; and Herse, Florian. Amaral LM often collaborated with Booz, George W; Campbell, Nathan; Elfarra, Amil T; Vaka, Venkata Ramana; Dechend, Raif; Muller, Dominik N; Jayaram, Aswathi; and Cottrell, Jesse N. The author visualization analysis based on CiteSpace (**Figure 4B**) shows that the size of nodes is determined by the number of citations, and co-citation relationships are represented by lines connecting nodes. As shown in **Table 3**, the University of Mississippi Medical Center (34 articles) is the largest contributor, followed by the Queens University, Canada (16 articles) and Harvard University (15 articles). Among the top 10 institutions in NK cells and PE research, two have a centrality ≥0.05: Chinese Academy of Sciences (0.13) and Harvard University (0.09).

**Table 2:** Top 10 authors in NK cells and PE.

Rank	Author	Record count	% of 525	Affiliations
1	Cornelius DC	25	4.76%	University of Mississippi Med Ctr JACKSON, MS, USA
2	Lamarca B	19	3.61%	University of Mississippi Med Ctr JACKSON, MS, USA
3	Ibrahim T	17	3.23%	Mansoura University Faculty of Pharmacy MANSOURA, EGYPT
4	Amaral LM	16	3.05%	University of Mississippi Med Ctr JACKSON, MS, USA
5	Campbell, Nathan	11	2.10%	University of Glasgow,Purdue University
6	Deer E	9	1.71%	University of Mississippi Med Ctr JACKSON, MS, USA
7	Cunningham MW	8	1.52%	University of North Texas Health Science Center Dept Physiol & Anat FT WORTH, TX, USA
8	Croy BA	7	1.33%	Queens University - Canada Queen's University Faculty of Health Sciences KINGSTON, ON, CANADA
9	Saito S	7	1.33%	University of Texas Medical Branch Galveston ; Brown University ; University of Toyama ; Datta Meghe Institute of Higher Education & Research (Deemed to be University); Jamia Hamdard University ; Institute for Research in Biomedicine - IRB Barcelona.et al.
10	Burke, SD	6	1.14%	Harvard Medical School

**Table 3:** Top 10 institutions in NK cells and PE.

Rank	Institution	Record count	% Of 525	Centrality
1	University of Mississippi (USA)	34	6.47%	0.03
2	Queens University, Canada (Canada)	16	3.05%	0.03
3	Harvard University (USA)	15	2.86%	0.09
4	Charite Universitatsmedizin Berlin (Germany)	11	2.10%	0.03
5	Free University of Berlin (Germany)	11	2.10%	0.03
6	Fudan University (China)	11	1.90%	0.01
7	Harvard University Medical Affiliates (USA)	10	1.90%	0.04
8	Chinese Academy of Sciences (China)	10	1.90%	0.13
9	Harvard Medical School (USA)	10	1.90%	0.03
10	Humboldt University of Berlin (Germany)	10	1.90%	0.02

Based on VOSviewer, we analyzed the institutional relationship (**Figure 4C**), six clusters were formed, with representative institutions within each cluster collaborating closely.

Group 1 includes the following institutions: univ of mississippi, yale univ, harvard med sch, eunice kennedy shriver natl in,which are closely cooperative. Cluster 2 includes the following institutions: toyama univ, univ Michigan, semmelweis univ, hungarian acad sci, eunice kennedy shriver natl in, michigan state univ, wayne state univ and univ n Carolina. Cluster 3 contains the following institutions: stanford univ, univ Cambridge, queens univ, univ Toronto, st marys hosp and Leiden univ, which are closely cooperative. The institutions included in Cluster 4 are st marys hosp, mt sinai hosp, univ Toronto, newcastle univ, univ Manchester, univ buenos aires, katholieke univ leuven, univ hong kong and univ pecs. The institutions included in Cluster 5 are fudan univ, Tongji univ, univ hong kang, shanghai jiao tong univ, shanghai key lab female reprod, south china univ technol, which are closely cooperative. Cluster 6 includes the following institutions: univ pecs, univ Manchester, janos szentagothai res ctr, univ med ctr hamburg Eppendorf.

In the institutional visualization analysis based on CiteSpace (**Figure 4D**), nodes with red outer rings and larger sizes represent key institutions involved in research related to NK cells and PE.

### 3.4 Disciplines and journals analysis

**Table 4** shows that Immunology, Reproductive biology, and Obstetrics & Gynecology are the top three disciplines in terms of publication volume. The remaining disciplines are Developmental Biology (8.76%), Biochemistry Molecular Biology (7.24%), Cell Biology (6.29%), Medicine Research Experimental (6.29%), Peripheral Vascular Disease (6.10%), Physiology (5.14%), Multidisciplinary Sciences (4%), Pathology (3.43%).

**Table 4:** Top 20 categories in NK cells and PE.

Rank	Record count	Web of Science categories	% of 525	Rank	Record count	Web of science categories	% of 525
1	171	Immunology	32.57%	11	18	Pathology	3.43%
2	162	Reproductive Biology	30.86%	12	17	Endocrinology Metabolism	3.24%
3	121	Obstetrics Gynecology	23.05%	13	15	Chemistry Multidisciplinary	2.86%
4	46	Developmental Biology	8.76%	14	14	Pharmacology Pharmacy	2.67%
5	38	Biochemistry Molecular Biology	7.24%	15	12	Genetics Heredity	2.29%
6	33	Cell Biology	6.29%	16	11	Medicine General Internal	2.10%
7	33	Medicine Research Experimental	6.29%	17	8	Biology	1.52%
8	32	Peripheral Vascular Disease	6.10%	18	8	Biotechnology Applied Microbiology	1.52%
9	27	Physiology	5.14%	19	7	Hematology	1.33%
10	21	Multidisciplinary Sciences	4%	20	6	Cardiac Cardiovascular Systems	1.14%

The analysis reveals that the *Journal of Reproductive Immunology* ranks first in publications related to NK cells and PE, followed by *Frontiers in Immunology*, *American Journal of Reproductive Immunology*, *Placenta*, *American Journal of Obstetrics and Gynecology*, and *International Journal of Molecular Sciences*. Among the co-cited journals, *Placenta* has the highest co-citation count, followed by *American Journal of Reproductive Immunology*, *Journal of Immunology*, and *American Journal of Obstetrics and Gynecology*. Seven of these journals had an IF > 10. These include *Journal of Experimental Medicine*, *Journal of Clinical Investigation, Nature Medicine, Science, Nature Reviews Immunology, Blood, Nature.*

**Figure 5:**
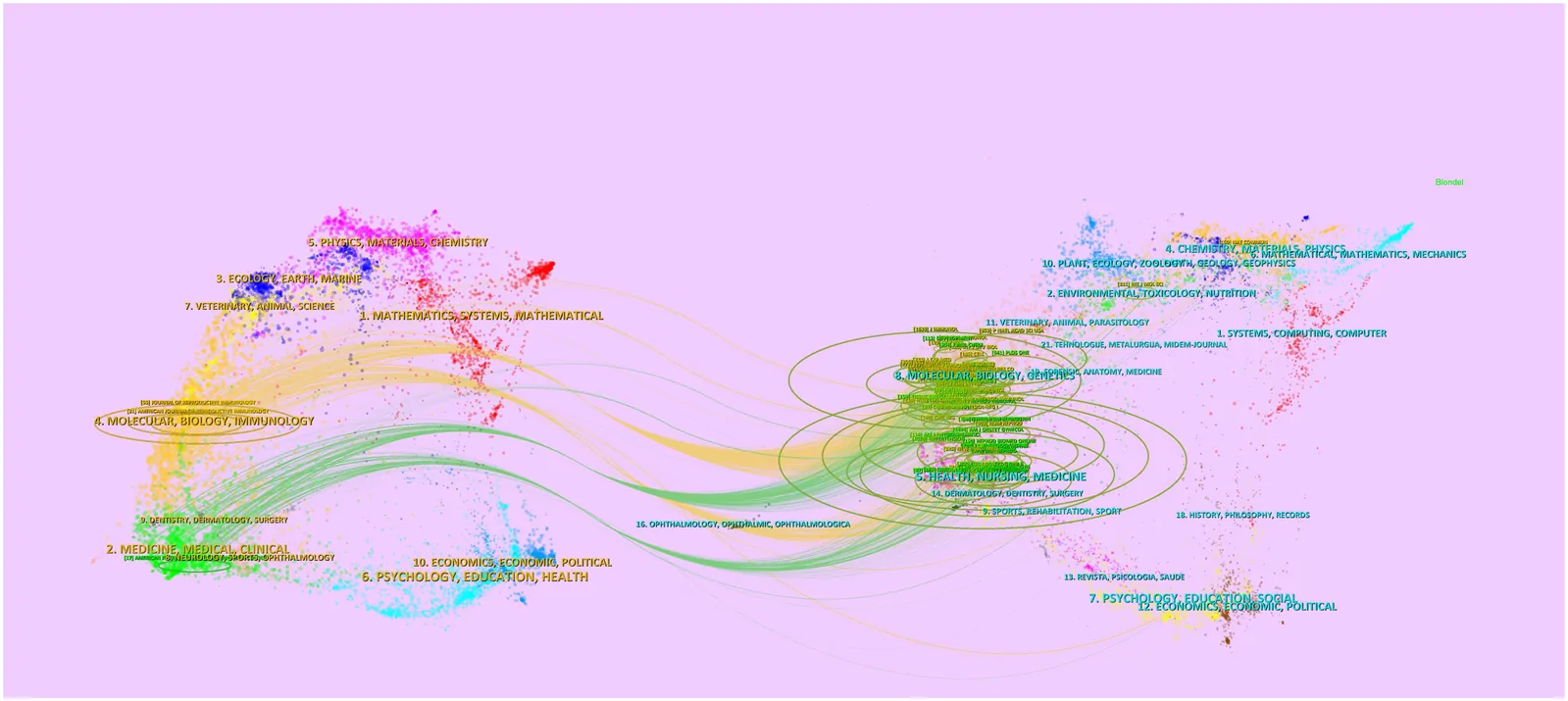
The dual-map overlay of journals in NK cells and PE.

**Figure 5** is a topic distribution analysis chart obtained using the dual-map overlay function, where the left side represents the citing part and the right side represents the cited part. Labels mark the involved disciplines, and colored paths indicate citation relationships. The orange path shows that journals in the Molecular/Biology/Genetics field and journals in the Health/Nursing/Medicine field are often cited by journals in the Molecular/Biology/Immunology field; the green path shows that journals in the Health/Nursing/Medicine field and journals in the Health/Nursing/Medicine field are often cited by journals in the Medicine/Medical/Clinical field.

### 3.5 Co-cited references and references analysis

**Figure 6:**
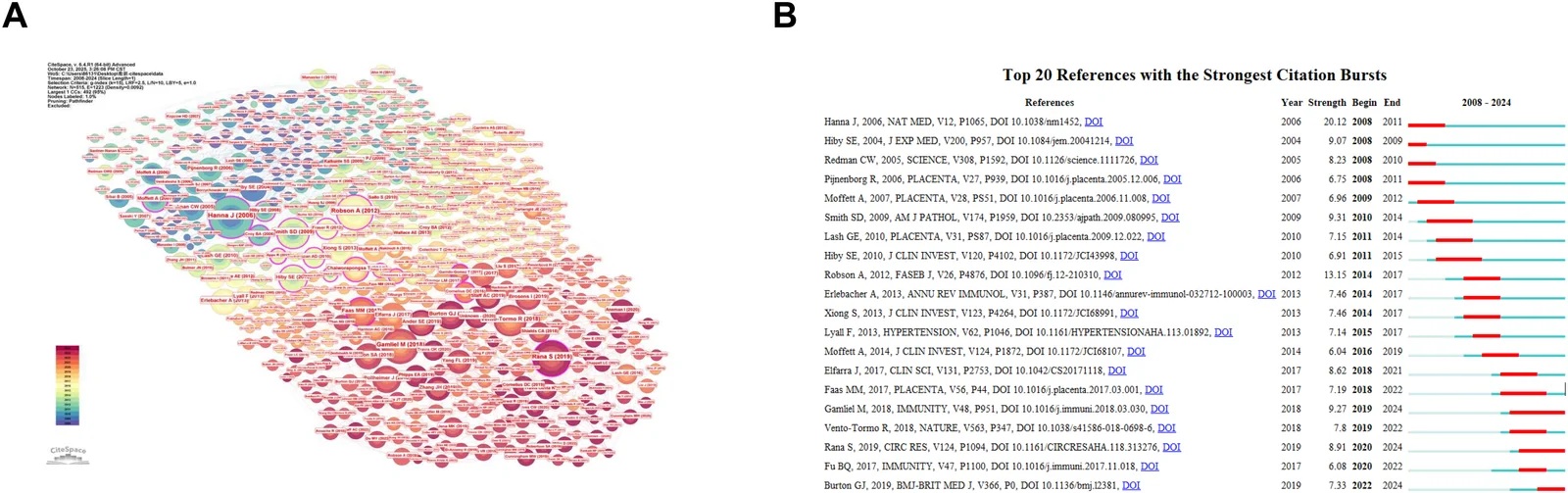
**(A)** References co-citation network in NK cells and PE. **(B)** Top 20 references with the strongest citation bursts in NK cells and PE.

**Figure 6A** shows the cited reference analysis of CiteSpace. **Table 5** lists the top 10 citations. Co-citation analysis indicates that the first-ranked document is “Decidual NK cells regulate key developmental processes at the human fetal-maternal interface” (16), followed by “Preeclampsia: Pathophysiology, Challenges, and Perspectives” (17). “Trained Memory of Human Uterine NK Cells Enhances Their Function in Subsequent Pregnancies” (18). **Figure 6B** displays the top 20 co-cited references. The strongest citation burst was “Decidual NK cells regulate key developmental processes at the human fetal-maternal interface” with a strength of 20.12.

**Table 5:** Top 10 cited references of publications in NK cells and PE.

Rank	Centrality	Title	Journal	Author	Year
1	0.12	Decidual NK cells regulate key developmental processes at the human fetal-maternal interface	Nature Medicine	Jacob Hanna (16)	2006
2	0.20	Preeclampsia : Pathophysiology, Challenges, and Perspectives	Circulation Research	Sarosh Rana (17)	2019
3	0.05	Trained Memory of Human Uterine NK Cells Enhances Their Function in Subsequent Pregnancies	Immunity	Moriya Gamliel (18)	2018
4	0.18	Uterine natural killer cells initiate spiral artery remodeling in human pregnancy	FASEB Journal	Andrew Robson (19)	2012
5	0.09	Single-cell reconstruction of the early maternal–fetal interface in humans	Nature	Roser Vento-Tormo	2018
6	0.17	Evidence for Immune Cell Involvement in Decidual Spiral Arteriole Remodeling in Early Human Pregnancy	American Journal of Pathology	Samantha D. Smith	2009
7	0.09	Pre-eclampsia: pathophysiology and clinical implications	British Medical Journal	Graham J Burton	2019
8	0.10	Uterine NK cells and macrophages in pregnancy	Placenta	Marijke M Faas	2017
9	0.06	Natural killer cells mediate pathophysiology in response to reduced uterine perfusion pressure	Clinical Science	Jamil Elfarra	2017
10	0.03	Regulation of Placental Extravillous Trophoblasts by the Maternal Uterine Environment	Frontiers in Immunology	Jürgen Pollheimer	2018

Jacob Hanna published the article titled “Decidual NK cells regulate key developmental processes at the human fetal-maternal interface” in Nature Medicine in 2006 (16). This article indicates that dNK cells regulate placental angiogenesis, decidualization, trophoblast invasion, and vascular remodeling through interactions between NK-specific receptors and ligands. This finding provides a new perspective for understanding the interaction between NK cells and trophoblast cells. In addition, it offers a theoretical basis for exploring the pathogenesis and therapeutic strategies of pregnancy-related diseases such as PE.

Sarosh Rana published the article titled “Preeclampsia: Pathophysiology, Challenges, and Perspectives” in *Circulation Research* in 2019 (17). Researchers such as Sarosh Rana have conducted in-depth investigations into the complications, pathogenesis, animal models, clinical diagnosis and treatment of PE, as well as the risk of future diseases caused by PE. The research team believes that placenta-mediated angiogenic imbalance is the pathological mechanism underlying the development of PE. The screening of biomarkers and novel targeted therapies will be of great significance for the diagnosis and treatment of PE in the future. Meanwhile, it is necessary to strengthen interventions for patients with PE to reduce the risk of long-term cardiovascular diseases.

Moriya Gamliel published the article titled “Trained Memory of Human Uterine NK Cells Enhances Their Function in Subsequent Pregnancies” in *Immunity* in 2018 (18). Moriya Gamliel and her team were the first to discover and confirm the existence of pregnancy-specific trained memory decidual natural killer cells (PTdNKs) in humans. They identified the uniqueness of these cells and expanded the boundary of knowledge about innate immune memory. They revealed that PTdNKs play an important role in placental development during repeated pregnancies. PTdNKs can reduce the risk of pregnancy-related diseases such as PE. In addition, this discovery provides potential targets for the clinical intervention of human pregnancy diseases related to poor placental development. Meanwhile, it offers a new perspective for understanding pregnancy-related immune regulation mechanisms.

Andrew Robson published the article titled “Uterine natural killer cells initiate spiral artery remodeling in human pregnancy” in the *FASEB Journal* in 2012. This article has a centrality of 0.18, indicating its influence (19). This article confirms for the first time in humans that uterine natural killer cells play an initiating role in spiral artery remodeling. Uterine natural killer cells can secrete angiopoietin-2 and matrix metalloproteinases. These substances destroy the tissue stability of vascular smooth muscle cells and the integrity of the extracellular matrix. By doing so, uterine natural killer cells fulfill the mission of initiating the early “trophoblast-independent” remodeling of spiral arteries in human pregnancy. This research finding reveals the molecular mechanism of vascular remodeling in early pregnancy. It also provides a new perspective for understanding the pathogenesis of pregnancy-related diseases such as PE.

### 3.6 Research hotspots and frontier analysis

**Figure 7:**
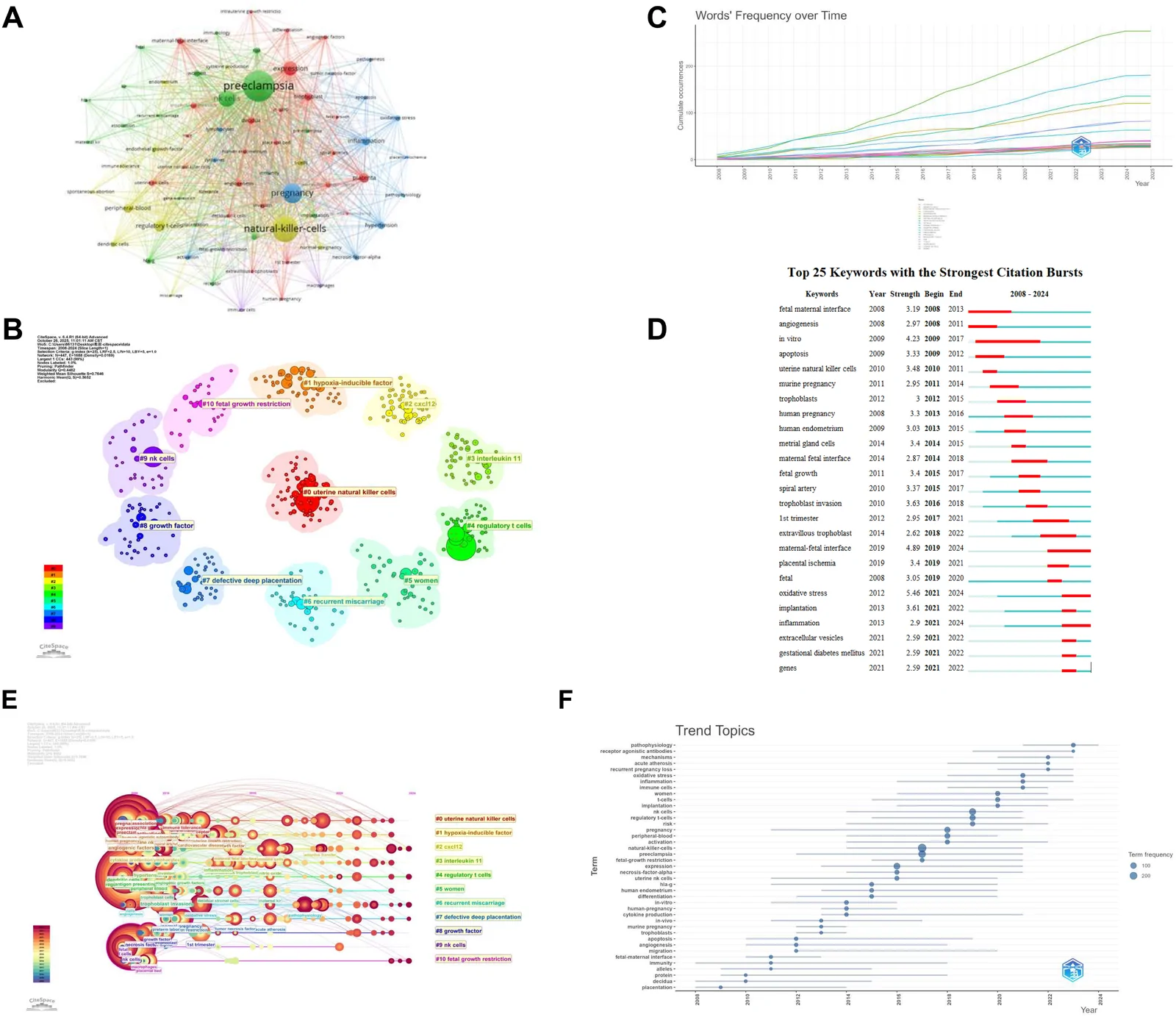
**(A)** The cluster of keywords in the studies of NK cells and PE. **(B)** Keyword Cluster Analysis. **(C)** The cumulative growth keywords (top 20). **(D)** Top 25 keywords with the strongest citation bursts. **(E)**Timeline viewer related to NK cells and PE. **(F)** The X-axis represents the year while the Y-axis is the cumulate occurrences of the keywords.

As illustrated in **Table 6**, besides natural killer cells (298) and preeclampsia (180), other high frequency keywords include, nk cells (148), expression (120), regulatory t-cells (89), pregnancy (85), peripheral-blood (66), t cells (41), trophoblast invasion (37), and risk (36). Among them, natural killer cells, preeclampsia, nk cells, expression, regulatory t-cells, and pregnancy were the top six frequencies, indicating they are research priorities. Keyword analysis based on the VOSviewer shows that these keywords form four color clusters in **Figure 7A**, representing four major research directions of NK cells and PE.

**Table 6:** Top 20 keywords in NK cells and PE.

Rank	Keywords	Occurrences	Centrality	Rank	Keywords	Occurrences	Centrality
1	natural killer cells	298	0.03	11	uterine nk cells	34	0.07
2	preeclampsia	180	0.07	12	women	34	0.03
3	nk cells	148	0.11	13	activation	33	0.15
4	expression	120	0.05	14	necrosis factor alpha	31	0.11
5	regulatory t cells	89	0.1	15	fetal growth restriction	30	0.07
6	pregnancy	85	0.08	16	dendritic cells	30	0.1
7	peripheral blood	66	0.07	17	oxidative stress	29	0.04
8	t cells	41	0.07	18	endothelial growth factor	27	0.08
9	trophoblast invasion	37	0.14	19	immune cells	25	0.04
10	risk	36	0.09	20	extravillous trophoblast	25	0.03

The red cluster mainly includes expression, *in-vitro*, trophoblast, differentiation, intrauterine growth restriction, maternal-fetal interface, trophoblast invasion, decidua nk cells, placenta, 1^st^ trimester, extravillous trophoblasts, placental bed, fetal-growth, spiral arteries.

The yellow cluster is mainly composed of natural-killer-cells, regulatory T-cells, dendritic cells, t-cells, normal-pregnancy, tolerance, peripheral-blood, spontaneous-abortion, endothelial-factor.

The green cluster mainly includes preeclampsia, nk cells, fetal, women, risk, HLA-G, receptor, placentation. The blue cluster mainly includes pregnancy, hypertension, implantation, activation, necrosis-factor-alpha, lymphocytes, oxidative stress, pathophysiology, immunity.

**Figure 7B** shows the results of keyword cluster analysis based on Citespace, which can be divided into four parts: related cells (#0 uterine natural killer cells, #4 regulatory t cells, #9 nk cells), related cytokines (#1 hypoxia-inducible factor, #2 cxcl12, #3 interleukin 11, #8 growth factor), pregnancy diseases (#6 recurrent miscarriage, #7 defective deep placentation, #10 fetal growth restriction), and gender (#5 women).

We conducted dynamic keyword analysis using the R package “bibliometrix” to explore hot trends. The annual growth rates of the top 20 keywords are presented in **Figure 7C**, and the analysis shows that some keywords have shown an upward trend since 2009. PE and NK cells showed a “J”-curve trend, while keywords such as normal pregnancy and expression show significant increases.

Based on the keyword co-citation network, **Figure 7D** presents the top 25 keywords with the strongest citation bursts in the field of NK cells and PE. Among them, oxidative stress (5.46) has the strongest citation burst, followed by maternal-fetal interface (4.89), *in vitro* (4.23), trophoblast invasion (3.63), implantation (3.61), uterine natural killer cells (3.48), placental ischemia (3.4), metrial gland cells (3.4), fetal growth (3.4) and spiral artery (3.37). Based on their start times of occurrence, angiogenesis, tolerance, apoptosis, HLA-G, uNK cells, *in vitro*, murine pregnancy, and fetal-maternal interface emerged earlier and were early research focuses. Maternal-fetal interface, oxidative stress, inflammation, implantation, and extracellular vesicles are current research frontiers in NK cells and PE, already in a burst phase.

The timeline view demonstrates the research hotspots centered on keywords and their dynamic change trajectories, reflecting the temporal characteristics within this research field and the evolution of hot keywords (20). Based on the cluster analysis of CiteSpace, **Figure 7E** intuitively displays the phased hotspots and development directions in NK cells and PE research from a temporal dimension. Cluster IDs are the numbered labels after clustering, such as #0, #1, #2, etc., and a larger cluster size indicates a greater number of member documents within the cluster. Based on the trend topic analysis using the R-base package “bibliometrix”, **Figure 7F** also presents the hot topics and directions in NK cells and PE research from a temporal perspective. Comparing the two datasets, research in 2008 mainly involved keywords such as immunity, protein, decidua, and placentation, while by 2024, the keywords had shifted to emerging hotspots including immune cells, oxidative stress, implantation, inflammation, and mechanism.

## 4 Discussion

### 4.1 General information

We used CiteSpace, VOSviewer, and the R package “bibliometrix” to analyze publications related to NK cells and PE. While reviewing research achievements and advancements, the analysis of data showed that the field of NK cells and PE began in 2008 and the overall trend was increasing. The higher citation rate indicates greater influence and higher research quality in this field, with citations increasing annually (**Figure 2**). Through statistical analysis of publications by country/region and institution, key countries/regions and research institutions with high publication volumes and significant influence in NK cell and PE research, as well as their collaborative relations were identified. The USA, China, and Canada are the main countries conducting research on NK cells and PE.

Among the top 10 institutions, four are from the USA, three from Germany, and the remaining three from China and Canada. The University of Mississippi Medical Center ranks first in both publication volume and H-index. Close collaboration between countries and institutions helps remove academic barriers and further advance research on NK cells and PE.

Top 10 contributors in terms of number of publications are: Cornelius DC (25, 4.76%), followed by Lamarca B (19, 3.61%) and Ibrahim T (17, 3.23%). Professor Cornelius DC and Professor Lamarca B rank as the top contributors to the h-index, as it is shown in **Table 2**. The h-index is also a quantitative measure. It can be used to gauge the quantity and the level of publications (21).

Professor Cornelius DC and Professor Lamarca B reviewed the role of NK cells in PE. In patients with PE, the number of pNK cells is increased, accompanied by enhanced cytotoxicity and activity, while their ability to synthesize and secrete VEGF is declined. In contrast, dNK cells are the most abundant immune cells at the maternal-fetal interface during early pregnancy and placenta formation. These cells are critical for secreting cytokines that promote the growth, differentiation, invasion of trophoblast cells, facilitate the remodeling of spiral arteries, and maintain the delicate balance of angiogenesis and maternal-fetal immune tolerance (6, 7).

Subsequently, Professor Ibrahim T and his colleagues investigated the role of NK cell responses in placental ischemia-induced hypertension and demonstrated that modulation of NK/T cell responses could attenuate hypertension and inflammatory reactions. NK cell activation contributes to inflammatory injury during pregnancy, and tumor necrosis factor-α (TNF-α) contributes to NK cell-mediated cytotoxic effects on placental and renal tissues, leading to excessive mitochondrial reactive oxygen species (ROS) production. Although mitochondrial respiration was not significantly altered, the decreased respiratory control ratio (RCR) suggested impaired mitochondrial efficiency, which may contribute to gestational hypertension and fetal death. Furthermore, 17-hydroxyprogesterone caproate (17-OHPC) was shown to regulate NK/T cell responses, alleviate inflammatory reactions, and improve placental ischemia-induced hypertension, providing potential therapeutic implications for PE (22–24).

Professor Saito S demonstrated that granulysin secreted by NK cells can induce apoptosis of extravillous trophoblasts (EVTs). Abnormal placental apoptosis has been implicated in the pathogenesis of PE. Additionally, NK cells express galectin-1 during normal late pregnancy, whereas fewer cells express this protein in non-pregnant women. Compared with normal pregnancy, the proportion of galectin-1-expressing NK cells in the peripheral blood of patients with PE is significantly reduced, which may trigger Th1/Th17-mediated pro-inflammatory immune responses and contribute to pregnancy-related disorders such as PE (25–27).

Analyzing the distribution of publication sources helps identify core journals for research related to NK cells and PE, providing references for scholars to explore better research directions. The results show that many of the involved journals are high-quality and high-impact, and these data will help future scholars select appropriate journals when submitting manuscripts. **Figure 5** indicates that articles from journals in the Molecular/Biology/Genetics field and journals in the Health/Nursing/Medicine field are often cited by journals in the Molecular/Biology/Immunology field and journals in the Medicine/Medical/Clinical field. This phenomenon indicates that current research on NK cells and preeclampsia mainly focuses on two directions: basic research and translational medicine.

**Table 4** shows that Immunology (171 articles, accounting for 32.57%) is the discipline with the highest number of publications, followed by Reproductive Biology (162 articles, accounting for 30.86%) and Obstetrics & Gynecology (121 articles, accounting for 23.05%). **Table 7** indicates that the *Journal of Reproductive Immunology* has the largest number of publications (51 articles, accounting for 9.71%), followed by *Frontiers in Immunology* (39 articles, accounting for 7.5%) and *American Journal of Reproductive Immunology* (29 articles, accounting for 5.52%). *Placenta*, *American Journal of Reproductive Immunology*, and *Journal of Immunology* are frequently co-cited. Among the top 20 journals by number of publications, 2 belong to the JCR Q1 category, with the *Journal of Clinical Investigation* (Impact Factor, IF = 13.6) ranking first. Among the top 20 journals by co-citation frequency, 9 fall into the JCR Q1 category, and 7 of these journals have an impact factor exceeding 10; the journal with the highest impact factor is *Nature Reviews Immunology* (IF = 60.9). JCR profiles are journal-specific metrics related to impact factor, ranking, and quartile categories (28).

**Table 7:** Top 20 journals and co-cited journals in NK cells and PE.

Rank	Record count	% of 525	Journal	IF	JCR	Co-cited Journal	IF	JCR
1	51	9.71%	*Journal of Reproductive Immunology*	2.9	Q3/Q3	*Placenta*	2.5	Q3/Q3
2	39	7.50%	*Frontiers in Immunology*	5.9	Q2/Q2	*American Journal of Reproductive Immunology*	2.4	Q3/Q4
3	29	5.52%	*American Journal of Reproductive Immunology*	2.4	Q3/Q4	*Journal of Immunology*	3.4	Q2/Q3
4	27	5.14%	*Placenta*	2.5	Q3/Q3	*American Journal of Obstetrics and Gynecology*	8.4	Q1/Q2
5	16	3.05%	*American Journal of Obstetrics and Gynecology*	8.4	Q1/Q2	*Proceedings of The National Academy of Sciences of The USA*	9.1	Q1/Q1
6	14	2.67%	*International Journal of Molecular Sciences*	2.5	Q3/Q4	*Biology of Reproduction*	3	Q2/Q3
7	13	2.48%	*Reproductive Sciences*	4.9	Q2/Q3	*Journal of Experimental Medicine*	10.6	Q1/Q1
8	11	2.10%	*Regulatory Integrative and Comparative Physiology*	2.3	Q3/Q4	*Human Reproduction*	6.1	Q1/Q2
9	9	1.71%	*Hypertension*	8.2	Q1/Q1	*Journal of Clinical Investigation*	13.6	Q1/Q1
10	9	1.71%	*Biology of Reproduction*	3	Q2/Q3	*Nature Medicine*	50	Q1/Q1
11	8	1.52%	*PLOS ONE*	2.6	Q3/Q3	*PLOS ONE*	2.6	Q3/Q3
12	8	1.52%	*Pregnancy Hypertension - An International Journal of Women's Cardiovascular Health*	2.9	Q4/Q4	*American Journal of Pathology*	3.6	Q2/Q3
13	7	1.33%	*Human Reproduction*	6.1	Q1/Q2	*Hypertension*	8.2	Q1/Q1
14	7	1.33%	*Molecular Human Reproduction*	3.5	Q3/Q3	*Human Reproduction*	6.1	Q1/Q2
15	6	1.14%	*Journal of maternal fetal neonatal medicine*	1.6	Q3/Q3	*Science*	45.8	Q1/Q1
16	6	1.14%	*American Journal of Pathology*	3.6	Q2/Q3	*Frontiers in Immunology*	5.9	Q2/Q2
17	5	0.95%	*Frontiers in cell and developmental biology*	4.3	Q2/Q2	*Nature Reviews Immunology*	60.9	Q1/Q1
18	5	0.95%	*Frontiers in Endocrinology*	4.6	Q2/Q3	*Blood*	23.1	Q1/Q1
19	5	0.95%	*Human Immunology*	2.2	Q4/Q4	*Journal of Leukocyte Biology*	3.1	Q2/Q4
20	5	0.95%	*Journal of Clinical Investigation*	13.6	Q1/Q1	*Nature*	48.5	Q1/Q1

In the first 10 references, those focusing on immune responses and angiogenesis are predominant, mainly because the pathogenesis of PE has not been fully understood. In the classical two-stage hypothesis, insufficient invasion of EVTs, leading to inadequate remodeling of spiral arteries (SPA), is considered the initiating factor in PE (29). Some pathological evidence shows that the patients with PE, the remodeling of SPAs at the placental implantation site is insufficient, and it often accompanied by reduced EVT invasion, collectively termed “shallow placental implantation” (30). Studies have indicated that shallow placental implantation can cause poor placental perfusion and hypoxia. Hypoxic placentas release various vasoactive substances and inflammatory cytokines, which further impair placental development and cause maternal vascular endothelial dysfunction, ultimately leading to PE (31–33). Among them, NK cells play a key role in the immune response and angiogenesis of PE. The high burst signals in the references indicate the intensity and time interval of attention (34). According to the top 20 references with the strongest citation bursts, the most cited publications in recent years are mainly related to the immune mechanisms and inflammation of PE.

### 4.2 Hotspots and frontiers

High-frequency keywords usually appear in the titles and abstracts of publications. They reflect the core research topics and thematic structure of a research field. Keyword co-occurrence analysis can reveal the relationships among research topics and their evolution over time (35). In this study, we integrated keyword co-occurrence, cluster, centrality, burst, and timeline analyses to systematically evaluate the research hotspots and developmental trends in NK cell-related PE research.

Keyword co-occurrence and cluster analyses identified four closely connected thematic modules: maternal-fetal interface immune regulation, inflammatory responses, immune cell interactions, and placental dysfunction. These themes highlight the close association among maternal-fetal immune dysregulation, inflammatory activation, placental dysfunction, and PE. Keyword centrality analysis showed that activation (centrality = 0.15), trophoblast invasion (centrality = 0.14), NK cells (centrality = 0.11), regulatory T cells (centrality = 0.10), and dendritic cells (centrality = 0.10) had relatively high network centrality. These keywords acted as important hubs connecting different research topics. Among them, trophoblast invasion showed high centrality, suggesting that it is a key link between immune regulation, placental development, and PE pathogenesis. It has also remained an important research focus in recent years. Keyword burst analysis identified oxidative stress and extracellular vesicles as the fastest-growing research topics in recent years. This trend suggests an increasing focus on molecular regulatory mechanisms and potential translational applications. However, keyword bursts only reflect a rapid increase in research attention during a specific period. They do not necessarily represent established scientific frontiers. Therefore, we consider these topics to be emerging research hotspots identified by bibliometric analysis rather than confirmed future research directions.

Timeline analysis further revealed the stage-specific evolution of research topics. From 2008 to 2013, studies mainly focused on maternal-fetal interface formation, decidual immune regulation, implantation, angiogenesis, and trophoblast invasion. Between 2013 and 2018, research shifted toward immune dysregulation in PE, including inflammatory responses, maternal-fetal immune tolerance, regulatory T-cell function, and fetal growth restriction. From 2019 to 2024, oxidative stress, extracellular vesicles, and inflammatory regulatory networks became major research topics. Overall, these findings indicate that NK cell-related PE research has gradually evolved toward complex multicellular interactions and molecular regulatory mechanisms.

#### 4.2.1 The maternal-fetal interface in PE

According to the keyword co-occurrence analysis, maternal–fetal interface-related keywords were mainly clustered in the red cluster, including maternal–fetal interface, decidual NK cells, trophoblast invasion, placenta, extravillous trophoblasts, and spiral arteries. These keywords formed a highly connected thematic module, suggesting that maternal–fetal interface immune regulation has remained a major research topic in NK cell-related PE research. Keyword cluster and thematic evolution analyses further showed that this topic has persisted throughout the development of the field, indicating sustained interest in the maternal–fetal immune microenvironment and its interactions with placental cells.

The maternal–fetal interface is the primary site of interactions between maternal immune cells and placental cells. Dysfunction of this interface can impair trophoblast invasion, disrupt spiral artery remodeling, and cause placental dysfunction, thereby contributing to PE development (36). Among immune cells, dNK cells are one of the predominant immune cell populations at the maternal–fetal interface. They regulate placental development through cytokines, growth factors, and angiogenic signaling (16, 37–39). The close association among decidual NK cells, trophoblast invasion, and spiral arteries in the co-occurrence network further suggests that dNK cells are key regulators linking immune regulation, placental vascular remodeling, and PE pathogenesis (40, 41).

#### 4.2.2 Inflammation in PE

According to the keyword co-occurrence analysis, inflammation-related keywords were mainly clustered in the green cluster, including preeclampsia, NK cells, fetal, women, risk, and HLA-G. These keywords formed a highly connected thematic module, suggesting that the interaction among inflammatory activation, immune regulation, and placental dysfunction represents an important component of NK cell-related PE research. Keyword cluster and thematic evolution analyses further showed that inflammation-related studies have continuously remained active throughout the development of this field. Inflammatory pathways have gradually emerged as an important research direction linking maternal–fetal immune regulation and placental dysfunction. In recent years, research has expanded toward complex regulatory networks involving immune cell interactions, inflammatory signaling pathways, and the placental microenvironment.

The importance of this inflammatory theme is consistent with previous studies. Impaired trophoblast invasion, abnormal spiral artery remodeling, and placental ischemia–hypoxia can induce inflammatory activation and disrupt maternal–fetal immune homeostasis. Therefore, the persistent presence of inflammation-related keywords reflects the close association between immune dysregulation and placental dysfunction. In addition, keyword burst analysis identified oxidative stress as a rapidly emerging research topic, suggesting that the interaction between inflammatory responses and oxidative damage may receive increasing attention in future studies (42).

The presence of HLA-G in the green cluster further highlights the importance of maternal–fetal immune tolerance in this research area. Previous studies have shown that abnormal HLA-G expression and altered immune cell functions may affect maternal–fetal immune balance and contribute to PE-associated placental dysfunction (42, 43). Furthermore, experimental models such as the reduced uterine perfusion pressure (RUPP) model have provided evidence supporting the involvement of inflammatory pathways in PE development. However, animal models cannot fully reproduce the complex immune microenvironment of the human maternal–fetal interface. Therefore, future studies using multiple experimental models are needed to further elucidate the role of inflammatory regulatory networks in NK cell-related PE (7, 44).

#### 4.2.3 Immune mechanisms and complications in PE

According to the keyword co-occurrence analysis, keywords related to immune regulatory mechanisms and PE-associated complications were mainly clustered in the blue and yellow clusters. These keywords included immune cells, inflammation, placenta, hypertension, pregnancy outcome, and maternal complications. They formed highly connected thematic modules in the co-occurrence network, suggesting that the interactions among immune dysregulation, placental dysfunction, and PE-related complications represent important research topics in NK cell-related PE studies. Keyword cluster and thematic evolution analyses further showed that immune-related research has gradually expanded toward complex regulatory networks involving maternal–fetal immune tolerance, immune cell interactions, inflammatory regulation, and maternal organ injury. In recent years, increasing attention has been paid to the effects of immune abnormalities at the maternal–fetal interface on trophoblast function, placental development, and PE-associated complications. These findings indicate that NK cell-related PE research is progressively moving toward multicellular interactions and immune regulatory network mechanisms.

##### 4.2.3.1 Immune mechanisms in PE

The presence of immune cell-related keywords in the cluster analysis indicates that alterations in immune cell composition and function are important topics in NK cell-related PE research. During normal pregnancy, the maternal immune system maintains a dynamic balance between fetal immune tolerance and immune defense. This immune homeostasis is essential for pregnancy maintenance and placental development (38, 45, 46).

Among these immune cells, dNK cells represent one of the major immune populations at the maternal–fetal interface. They participate in the regulation of trophoblast function and placental development. Previous studies have shown that abnormal NK cell function and alterations in other immune cell populations may disrupt maternal–fetal immune homeostasis and contribute to PE development (7, 36, 43, 47).

##### 4.2.3.2 Complications in preeclampsia

Keyword cluster analysis showed that hypertension, pregnancy outcome, and maternal complications were mainly located in thematic modules related to PE clinical manifestations and disease outcomes. These findings suggest that NK cell-related immune abnormalities may not only affect local immune regulation but also contribute to PE-associated complications and long-term maternal health risks.

Thematic evolution analysis further indicated that research in this field has gradually focused on the relationship among immune dysregulation, placental dysfunction, and maternal clinical outcomes. Previous studies have demonstrated that placental abnormalities, ischemia–hypoxia conditions, and immune activation are closely associated with hypertensive disorders of pregnancy and adverse pregnancy outcomes. In addition, women with a history of PE have an increased risk of cardiovascular disease later in life, suggesting that PE-associated immune abnormalities and placental dysfunction may have long-term effects on maternal health (48–50).

### 4.3 Future frontiers

Based on keyword co-occurrence analysis, cluster analysis, centrality analysis, keyword burst analysis, and thematic evolution analysis, we identified the potential research frontiers that have emerged in the field of NK cell-related PE research in recent years. It should be noted that keyword bursts and thematic evolution in bibliometric studies mainly reflect changes in research attention and do not necessarily represent confirmed biological mechanisms or clinical directions. Therefore, the following discussion aims to interpret the potential reasons underlying the formation and development of these research topics based on bibliometric findings, rather than providing an independent review of their biological mechanisms.

#### 4.3.1 Extravillous trophoblast

Based on keyword centrality analysis, keyword cluster analysis, and timeline evolution analysis, trophoblast invasion was identified as an important connecting keyword in NK cell-related PE research, with relatively high network centrality (centrality = 0.14). In addition, EVT-related keywords, including trophoblast invasion, placenta, spiral arteries, and extravillous trophoblasts, formed a highly connected thematic module in the keyword co-occurrence network. Timeline analysis showed that early studies mainly focused on maternal–fetal interface formation, trophoblast migration, and invasion. In recent years, research attention has gradually shifted toward the dynamic interactions between immune cells and trophoblasts. These findings suggest that EVT-related research not only focuses on trophoblast invasion but also reflects the close relationship between maternal–fetal immune regulation and placental function.

EVTs are derived from cytotrophoblasts (CTBs) and represent a key trophoblast population involved in placental invasion and spiral artery remodeling. By migrating into decidual tissues and maternal spiral arteries, EVTs promote vascular remodeling and establish an appropriate maternal–fetal circulation to support fetal development (51–53). Therefore, impaired EVT function may lead to abnormal placental vascular remodeling and contribute to PE development (54, 55). Consistent with the bibliometric findings, the high centrality of trophoblast invasion indicates that it is not only a critical process in placental development but also an important link connecting immune regulation, placental dysfunction, and PE pathogenesis.

Further studies have shown that EVT function is regulated by the maternal–fetal immune environment. As major immune cells at the maternal–fetal interface, dNK cells interact with EVTs through receptor–ligand pathways, such as the KIR–HLA-G pathway, and regulate EVT migration, invasion, and spiral artery remodeling. Therefore, abnormal dNK–EVT interactions may represent a key mechanism linking NK cell dysfunction, impaired trophoblast invasion, and PE development. In addition, EVTs contribute to maternal–fetal immune tolerance. For example, EVTs can express TGF-β1, which promotes Treg differentiation and helps maintain local immune homeostasis (56, 57). These findings are consistent with the immune regulation-related themes identified by keyword clustering analysis.

#### 4.3.2 Implantation

Based on keyword co-occurrence analysis, keyword cluster analysis, and thematic evolution analysis, implantation was identified as an important research theme in NK cell-related PE studies. In the keyword co-occurrence network, implantation formed a highly connected thematic module with maternal–fetal interface, trophoblast invasion, decidual NK cells, and placenta. These findings suggest that implantation is an important biological process linking maternal–fetal immune establishment, trophoblast function, and early placental development. Timeline evolution analysis showed that studies from 2008 to 2013 mainly focused on embryo implantation, maternal–fetal interface formation, trophoblast migration and invasion, and local immune regulation. In recent years, research attention has gradually shifted toward the dynamic interactions between immune cells and trophoblasts and their roles in PE development. Therefore, the persistent appearance of implantation as a keyword reflects the transition of this field from focusing on early pregnancy establishment to exploring the relationship between maternal–fetal immune regulatory networks and placental dysfunction.

Implantation is a critical early process for pregnancy establishment and placental formation. Abnormal implantation may affect EVT migration and invasion, spiral artery remodeling, and subsequent placental function (58–62). Based on the bibliometric findings, dNK cells were identified as key connecting nodes linking implantation, trophoblast invasion, and spiral artery remodeling. dNK cells regulate EVT function through the secretion of cytokines and growth factors and contribute to the establishment of maternal–fetal immune tolerance. In addition, other immune cell populations, including macrophages and dendritic cells, participate in tissue remodeling and immune regulation at the early maternal–fetal interface, thereby maintaining a suitable microenvironment for implantation (63).

#### 4.3.3 Extracellular vesicles

Based on keyword burst analysis, extracellular vesicles (EVs) represent one of the rapidly emerging research topics in NK cell-related PE studies in recent years and show a significant keyword burst. Combined with thematic evolution analysis, these findings indicate that research in this field has gradually shifted from traditional immune cell characterization toward the investigation of intercellular communication and molecular regulatory networks. Therefore, EVs, as important mediators of information transfer, may serve as key regulatory nodes connecting immune cells, trophoblast cells, and the maternal–fetal interface microenvironment.

EVs are nanoscale extracellular particles enclosed by lipid bilayer membranes. They carry various bioactive molecules, including miRNAs, mRNAs, and proteins, and regulate recipient cell functions through cellular uptake (64–67). At the maternal–fetal interface, both immune cells and trophoblast cells can release EVs, thereby participating in intercellular communication. EVs provide a new perspective for understanding the interactions between NK cells and trophoblast cells.

In recent years, the role of EVs in PE development has received increasing attention. Placenta-derived EVs can enter the maternal circulation and participate in immune regulation, inflammatory responses, and placental function maintenance. Due to their stability and ability to carry molecular information, EVs have potential value as biomarkers for early diagnosis and risk prediction of PE (68–72). For example, increased levels of placental alkaline phosphatase (PLAP) and altered expression of miR-153-3p and miR-325-3p in plasma EVs from patients with PE suggest that EV-associated molecules may serve as potential biomarkers for disease monitoring (73).

#### 4.3.4 Oxidative stress

According to keyword burst analysis, oxidative stress showed the strongest increase in research attention among NK cell-related PE studies. It showed the highest burst strength (Strength = 5.46) and remained active from 2021 to 2024. Combined with thematic evolution analysis, these findings indicate that research in this field has gradually shifted toward inflammatory responses, oxidative damage, and multicellular interaction networks. Therefore, oxidative stress may represent an important regulatory link connecting immune activation, placental dysfunction, and PE progression.

Oxidative stress is characterized by increased production of reactive oxygen species (ROS) that exceeds antioxidant defense capacity, resulting in cellular damage (74). During normal pregnancy, the placenta maintains a dynamic redox balance. However, excessive ROS accumulation in PE leads to placental oxidative injury. Syncytiotrophoblasts (STBs) are directly exposed to maternal circulation and are major targets of oxidative damage. Studies have shown that STBs in PE placentas exhibit structural abnormalities, mitochondrial dysfunction, and reduced antioxidant capacity (75, 76). In addition, oxidative stress may regulate EV-mediated intercellular communication, which is consistent with the increasing research interest in EVs (42). Oxidative stress can also promote inflammatory responses, induce apoptosis, and impair placental vascular function, thereby contributing to PE development (77). For example, sFlt-1-mediated inhibition of VEGF signaling and endothelial dysfunction may promote abnormal placental vascular function, forming a vicious cycle between oxidative stress and anti-angiogenic imbalance (44).

In recent years, therapeutic strategies targeting oxidative stress-related pathways have received increasing attention. Although traditional antioxidants have shown limited efficacy in PE prevention, molecules with antioxidant and immunomodulatory properties remain potential therapeutic targets (78–80). Future studies should integrate multi-omics approaches and functional experiments to further clarify the specific role of oxidative stress in the interaction network between NK cells and trophoblast cells.

## 5 Limitations

This study has several limitations. First, the included publications were mainly retrieved from the WoSCC and PubMed databases. Due to differences in journal coverage, indexing methods, and bibliographic structures among databases, different databases may identify partially different sets of publications even when similar search strategies are applied. In addition, other databases, such as Scopus, were not included in the present study. Therefore, some relevant publications may have been missed, which may have affected citation analysis, collaboration network analysis, and the identification of research trends. Second, although a comprehensive search strategy was applied, differences in terminology, indexing methods, and database-specific retrieval mechanisms may influence literature identification. Although the major search terms and retrieval strategies have been reported, some studies using alternative terms may not have been identified. Third, only English-language publications were included in this study, which may introduce language bias and potentially exclude relevant studies published in other languages. Fourth, different bibliometric analysis tools, including VOSviewer, CiteSpace, and bibliometrix, use different algorithms and parameter settings for network construction and visualization. Therefore, variations among analytical platforms may influence the presentation and interpretation of research trends. Finally, citation-based indicators may be influenced by publication age, citation practices, and differences among research fields. Therefore, highly cited publications do not necessarily represent the most scientifically significant studies, and bibliometric findings should be interpreted together with biological and clinical evidence.

## 6 Conclusion

Through bibliometric analysis of publications on PE and NK cells, we analyzed publication trends, thematic developments, and emerging research hotspots for future studies, and provided potential collaborators and foundational insights for researchers in this field. Current research hotspots primarily focus on oxidative stress, the maternal-fetal interface, inflammation, EVs, implantation, and EVTs. This work represents the first systematic bibliometric analysis of NK cells and PE, offering an objective and comprehensive summary for researchers in this field and serving as a valuable reference for future investigations.

## 7 Future perspectives and implications

This study summarized the research trends and emerging directions in NK cell-related PE research using bibliometric methods. Keyword co-occurrence analysis, cluster analysis, burst analysis, and thematic evolution analysis showed that research in this field is gradually shifting toward the integrated study of the maternal–fetal immune microenvironment, multicellular interactions, and placental regulatory networks. Future studies may combine single-cell RNA sequencing, spatial transcriptomics, and multi-omics approaches to further investigate the dynamic interactions between NK cells and trophoblast cells, and to identify key cell populations and molecular pathways involved in PE development. In addition, placental organoids, placenta-on-a-chip models, and gene-editing technologies may provide more physiologically relevant models of the human maternal–fetal interface to validate critical regulatory mechanisms. Overall, this study summarizes the knowledge structure and developmental trends of NK cell-related PE research and provides valuable insights for future investigations in this field.

## Data Availability

The original contributions presented in the study are included in the article/supplementary material. Further inquiries can be directed to the corresponding authors.
